# Teaching the principles of least-cost poultry feed formulation utilizing the Solver function within a computer software workbook

**DOI:** 10.1016/j.psj.2024.103636

**Published:** 2024-03-08

**Authors:** G.M. Pesti, M. Choct, P.V. Chrystal, M.R. Bedford, A.F. Moss

**Affiliations:** ⁎ The Poultry Hub Australia, CJ Hawkins Homestead, University of New England, Armidale New South Wales, 2351, Australia; † Poultry Research Foundation, The University of Sydney, Camden, NSW 2570, Australia; ‡ Aviagen Australia, Goulburn, New South Wales, 2580, Australia; § AB Vista, Marlborough, Wiltshire SN8 4AN, United Kingdom; # Homestead building, School of Environmental and Rural Science, University of New England, Armidale, NSW, 2351, Australia

**Keywords:** feed formulation, linear programming, tertiary Instruction

## Abstract

A Microsoft Excel workbook, User-Friendly Feed Formulation with Data from Australia (UffdAu.xlsm), has been developed for teaching feed formulation techniques to tertiary level, university students. It runs under both Microsoft Windows and Apple iOS operating systems. The example ingredient composition matrix is based on the Australian Feed Ingredient Database to illustrate the biological and econometric principles of least-cost feed formulation. The nutrient data are based roughly on recent primary breeder company recommendations. The workbook is easily adapted to appropriate ingredients, nutrients, and prices most relevant to the students, wherever it is used. The workbook uses the linear routines of Excel's Solver add-in under the Data heading in the header Ribbon. There is a worksheet illustrating how to adapt non-linear responses such as exogenous enzymes to typical linear models using a step function. Additional worksheets illustrate how proximate analysis can be interpreted in modern analytical chemistry terms and, how various feed energy measures are related to feed composition. UffdAu.xlsm is available free of charge from the Poultry Hub Australia website (https://www.poultryhub.org).

## INTRODUCTION

The mechanics or process of feed formulation has changed little since the 1950s. The nutritionist collects and combines 4 sets of data: the nutrient composition of the ingredients available, the minimum and maximum acceptable nutrient levels in the feed, the prices of the ingredients, and the minimum and maximum raw material bounds (Pesti and Choct 2023). Computers are then used to find the least-cost combination of ingredients meeting the nutrient specifications. Oviedo-Rondon (2014) presented a very insightful list of reasons why this process is inefficient, outdated, and yet remains the most utilized mathematical algorithm globally.

Teaching the basics of feed formulation is still needed to equip students with the knowledge of the present system and how it may be improved and updated. Poultry meat and egg production is basically turning feed into meat and eggs. That makes feed formulation a very important skill for workers in the poultry industries. Microsoft Excel workbooks can accomplish the mechanics of feed formulation, by solving least-cost, linear feed formulation using Simplex Methodology (Dantzig, 1963). Since it is practically ubiquitous on laptop computers and already used by many instructors and students, it is an ideal instructional media basis for studying feed formulation.

User-Friendly Feed Formulation (Pesti and Miller, 1988) was originally written in Fortran and updated to run in Excel under Microsoft Windows. It has been used at many universities around the world (United States, Australia, Brazil, New Zealand, etc.) because of the ease of customization for different feedstuffs and species. A recent unsolicited email: *“dear sir, I still using your UFFDA till now. I am teach Indonesian poultry farmer by Facebook. Thank you regards, Antimon Ilyas PhD Jakarta, Jakarta Raya, Indonesia .* The current version, User-Friendly Feed Formulation with data from Australia (UffdAu.xlsm, or UffdAu) was written to run on both Windows and Apple Computer's MacOS operating systems (Apple, 2019; Bott and Stinson, 2019). Several worksheets have been added to the current version to assist students to understand how feed formulation can be made more efficient and updated.

## MATERIALS AND METHODS

To illustrate the principles, practices and possibilities of feed formulation, UffdAu, primarily uses 12 ingredients (wheat, sorghum, barley, corn, soybean meal (Brazil), soybean meal (local), canola meal (solvent extracted) canola meal (old pressed), lupins, peas, meat meal and meat and bone meal) from the Australian Feed Ingredient Database (**AfiD**, Moss 2020). AfiD was compiled from Australian producers from either locally grown or imported ingredients (Moss, 2020). There are 3 main worksheets in the UffdAu workbook that combine the ingredient composition dataset, nutrient and ingredient bounds (minimums and maximums), and ingredient prices to solve for the least-cost solution. Some additional worksheets illustrate how formulation data is derived from raw data. Others present the results in tabular and graphic formats to print and visualize formulation results. It is possible for instructors to easily adapt the ingredient and nutrient matrices, and costs, to local conditions and make the examples fully relevant to their students.

The actual matrix that is solved is on the “Formulate” worksheet, hidden from view. This matrix is built from the “ACTIVE INGREDIENT COMPOSITION MATRIX” section of the “Ingredients” worksheet and the “CURRENT SPECIFICATION” section of the “Nutrients” worksheet. Results from there are transferred to the visible section of the “Formulate” worksheet and re-formatted to improve their presentation.

### The Basic Feed Formulation Problem

UffdAu can solve any (feasible) linear feed formulation problem, regardless of complexity. The “Active Ingredient composition matrix” that is solved contains up to 25 ingredients and up to 34 nutrients. An almost unlimited number of ingredients and nutritional specifications can be stored and copied into the active sections of the “Ingredients” and “Nutrients” worksheets, respectively.

Instructors are able to begin with very simple (or traditional) problems and then add in features like ratios between nutrients, digestibility coefficients, net energy equations, and, nonlinear responses to exogenous enzymes. Any features added to commercial linear formulation programs should be possible with UffdAu's Excel formulation model.

### Understanding Feed Composition

**Figure 1:**
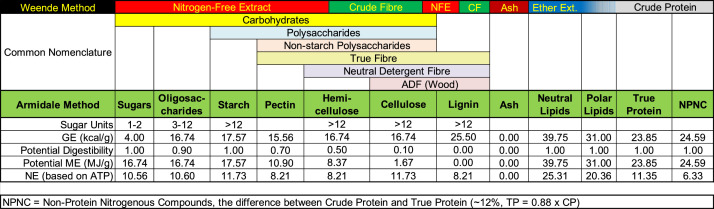
A comparison of the proximate analysis (Weende Method) and astute analysis (Armidale Method) and the potential of the various feed components to provide energy for animals. The fade from ether extract to crude protein in the first row depicts the potential double counting of phospholipids as ether extract and Crude Protein.

**Figure 2:**
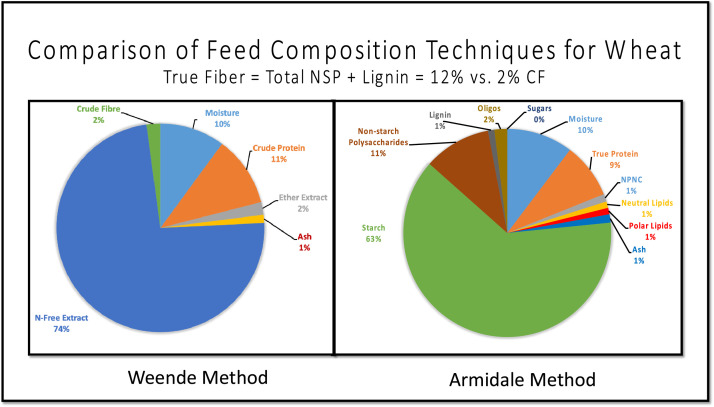
A comparison of the composition of an Australian wheat sample using proximate (Weende) and astute (Armidale) description methods.

Modern animal nutritionists describe feed ingredients and, most notably their energy contents, using proximate analysis methods developed in the city of Weende in the Hanover Kingdom in 1866 (Severe, 2022); described as proximate analysis, or the Weende Method. The “understanding feed composition chart” (Figures 1 and 2) or “Armidale Method” was originally designed to compare 19th Century feed terminology to modern, better understood 21st Century chemistry. Basically, what was labeled “Nitrogen-free Extract” is now known to be sugars, oligosaccharides, starches, pectin, some hemicellulose, some lignin, and some cellulose. Crude protein contains true protein (amino acid polymers), DNA, RNA, and any other nitrogen-containing compounds including the phospholipids (which contain choline, serine, and other compounds). There are many challenges when describing ingredients in terms of the Weende Method, eloquently explained over 40 years ago (Wardeh, 1981). The true nature of proteins, carbohydrates, and lipids were not known when the method was originally described. Since 1866, nutritionists have developed an understanding of problems that contribute to Weende Method inefficiencies. For example, the nitrogen-free extract (**NFE**) contains many types of compounds with differing energy contents, digestibilities and rates of absorption. Additionally, only a portion of crude fibre (**CF**) is hemi-cellulose, cellulose and lignin, whilst some of the ash is volatile under typical assay conditions. Also, ether extract contains mainly neutral lipids and few polar lipids. Petroleum ether has replaced ether in some laboratories for modern proximate analysis, so what is often referred to as “ether extract” may now contain more nonpolar lipids than before (AAFCO, 2014). The polar lipids may contain large amounts of phospholipids that include choline and serine. These lipids are also considered part of crude protein (**CP**) when estimated from ingredient nitrogen content (if measured before lipids have been extracted). Phosphatidylserine, while not part of true protein in feed ingredients, may be available for protein synthesis by animals. Similarly, phosphatidyl choline is oxidized to betaine by birds and animals and then to glycine, which is thereby potentially available for protein synthesis by animals. The non-protein nitrogenous compounds (**NPNC**) composition and quantities are not well known for feeds. Their characteristics are assumed to be the same as true protein. Notably, the Armidale Method could be a long-needed replacement for the 19th Century Weende method. Worksheets are included in UffdAu demonstrating comparison between historic and modern methodology.

### Energy Determinations And Ingredient Composition

Apparent metabolizable energy (**AME**) is presently the primary unit for characterizing feed ingredients for poultry. The first widely used method of estimating the AME_n_ (nitrogen corrected AME) based on proximate analysis is a series of equations published by the European Branch of the World's Poultry Science Association (1989). These equations continue to be used sometimes in feed formulation. UffdAu includes worksheets illustrating how the WPSA (1989) equations are used to estimate the AME_n_ of feed ingredients from their proximate analyses (and starch, in the case of barley). Worksheets are also included showing how metabolizable energy (**ME**) and net energy (**NE**) can be calculated based on Aridale Method composition and digestibility data (Figure 1). ME estimations are based on physiological fuel values (Kleiber, 1975). NE estimations are based on the ability of nutrients to generate ATP (Livesey, 1984; Coles et al., 2013; Van der Klis et al., 2010). Mateos et al. (2019) detailed shortcomings of all the common methods of estimating AME currently used. UffdAu includes several approaches that may be included (by students) in improved models in the future. Much of the ingredient composition data needed for the implementation of the Armidale Method (Figure 1) is already available because ruminant nutritionists need to understand different types of fiber (Bach Knudsen and Nygaard Lærke, 2018). Specific energy contents for the various fractions will improve the overall estimation of the energy in each feed ingredient. Such values have already been developed for human nutrition (FAO, 2003). A worksheet is included in UffdAu demonstrating how feed composition is related to common calculations of feed energy for students to compare.

### Adding Non-Linear Responses to Linear Models, Responses to Exogenous Enzymes

Enzymatic digestion from both endogenous and exogenous enzymes follows Michaelis-Menten enzyme kinetics (Johnson and Goody 2011). Many nutrition students have recently studied biochemistry and so are familiar with Michaelis-Menten enzyme kinetics and the linear Lineweaver-Burk transformation used to derive it (Lineweaver and Burk, 1934). An UffdAu worksheet uses the Lineweaver-Burk equation to demonstrate how the nonlinear response to exogenous phytase (Shirley and Edwards, 2003) can be added to formulation problems. A worksheet is included in UffdAu demonstrating how a step function can be used to approximate a nonlinear response in a linear model. The more steps added to the function, the better it will approximate the true Michaelis-Menten enzyme kinetics. Understanding the kinetics, the diminishing returns phenomena, is key for students to apply economics to much technical response data.

### Digestible True Protein Levels

UffdAu includes a worksheet showing calculations for digestible protein from digestible amino acid data. This worksheet also estimates specific nitrogen to protein conversion factors, calculated from total amino acid data (Sriperm et al., 2011). Calculations for glycine that can be formed in vivo from dietary choline and betaine are also included on an equimolar basis with the assumption that this conversion is 100% efficient.

### Non-Essential Amino Acid “Requirements”

Nutritionists have long considered the conundrum of including “requirements” for the sum of the non-essential amino acids in feed formulation models. The problems are firstly, that the nonessential amino acids can be interconverted, and secondly, that any excesses of the non-essential amino acids can be converted into various non-essential amino acids. For instance, the amino group from excess phenylalanine can be used for de novo synthesis of alanine. It would be possible to calculate the sum of the excess essential and total non-essential amino for each diet, but it is easier to simply calculate the digestible lysine (**dLys**) requirement to total protein ratio. Alhotan and Pesti (2016) demonstrated a strong relationship between dLys requirements and total digestible true protein across 29 recent broiler chicken experiments. They estimated that the dLys:dTP should be approximately 1:16.67 to maximize the feed utilization efficiency of broiler chickens. UffdAu includes calculations to assure a minimum ratio of dLys to true protein level in formulation problem solutions, thus assuring adequate amounts of the total non-essential amino acids whilst limiting excess essential amino acids.

### Nonprotein Nitrogen as Part of “Crude Protein”

Nonprotein nitrogenous compounds (**NPNC**) “also include numerous substances such as nucleic acids, amines, urea, ammonia, nitrates, nitrites, phospholipids, nitrogenous glycosides, etc. The fraction of non-alpha-amino nitrogen is highly variable for a given protein source, depending on the production process and the degree of purification of the protein source” (Mariotti et al., 2008). However, there remains a paucity of data on the entire nature of the NPNC (Imafidon and Sosulski, 1990a,b), the amounts in various ingredients, their energy contents, digestion, or absorption. Furthermore, there are some reports of NPNC fractions being valuable dietary supplements for broilers (Mohamed et al, 2020; Jung and Batal, 2012). There has generally been little interest in the NPNC of feed ingredients, so precise factors for the conversion of the various NPNC are not known. UffdAu includes calculations demonstrating the amount of amino acids that may be derived from 2 NPNC compounds (choline and betaine).

### Key Worksheets in the UffdAu Workbook

**Figure 3:**
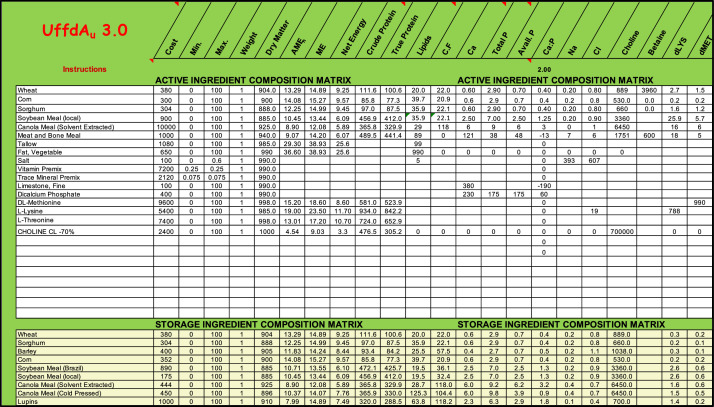
A portion of the UffdAu.xlsm “Ingredients” worksheet. To be used in a formulation problem, rows from the STORAGE INGREDIENT COMPOSITION MATRIX are copied to the ACTIVE INGREDIENT COMPOSITION MATRIX.

The Ingredients Worksheet has an area for ingredient composition data to be used in the current problem that is to be formulated (Figure 3). Beneath the Active Ingredient Composition Matrix box are many more ingredients, or entire feed ingredient lists, that can be copied into the Active Ingredient Composition Matrix section when needed.

**Figure 4:**
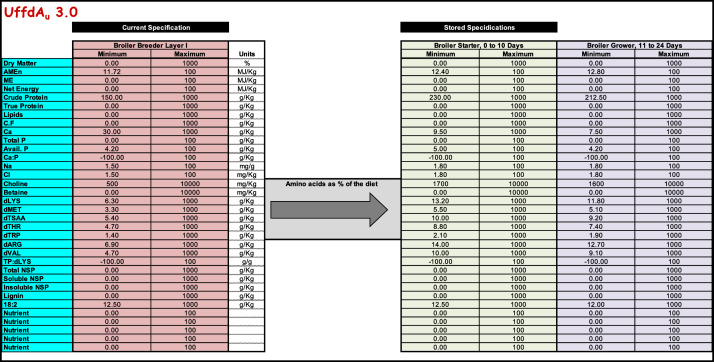
A portion of the UffdAu.xlsm “Nutrients” worksheet where requirements and maximums are specified. The data in the “Current Specification” section is the data that will be used when invoking the Solver routine from the Formulate Worksheet.

The Nutrients Worksheet has an area containing the Current Specification (Figure 4). That is where the nutrient requirements are found for the current problem to be solved. Many more feed restrictions can be stored to the right of the Current Specificiation. There are 2 versions of each feed that can be copied into the Current Specification cells. One has amino acid requirements as g/kg of diet. The other has the amino acids as a proportion of dLys content. This way of specifying amino acids in this manner may be used when keeping the amino acids in balance is thought necessary.

**Figure 5:**
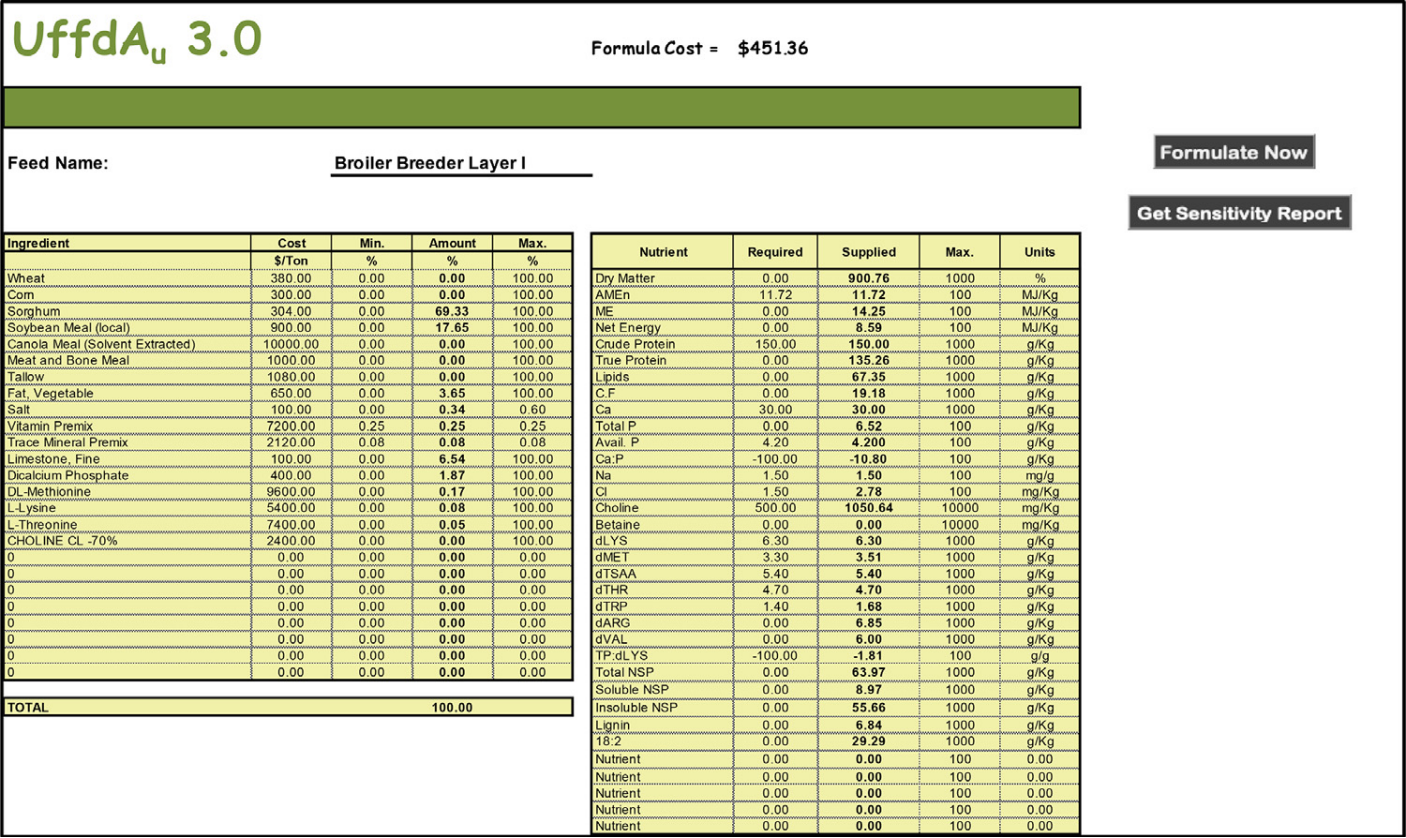
A portion of the UffdAu.xlsm “Formulate” worksheet. The solver add in is run from this worksheet and results are displayed.

The Formulate Worksheet is where the Solver routine of Excel is invoked (by clicking on the <Formulate Now> button) to find the least-cost solution (Figure 5). If parametric cost ranging is important, the <Get Sensitivity Report> button can open a new worksheet from which shadow prices can be calculated. Changes should not normally be made to the Formulate worksheet as they will be permanent and break links to other worksheets.

The Feed Spec Worksheet is another presentation of the formulation results to print or store your solutions. Column P contains the ingredient and nutrient levels of the last feed formulated for copying to other columns to be stored.

The Mixing Sheet Worksheet calculates how much of each ingredient is needed for a specified batch size of the last formulation result.

The Graphs Worksheet presents how each nutrient compares to the requirement specified for it.

**Figure 6:**
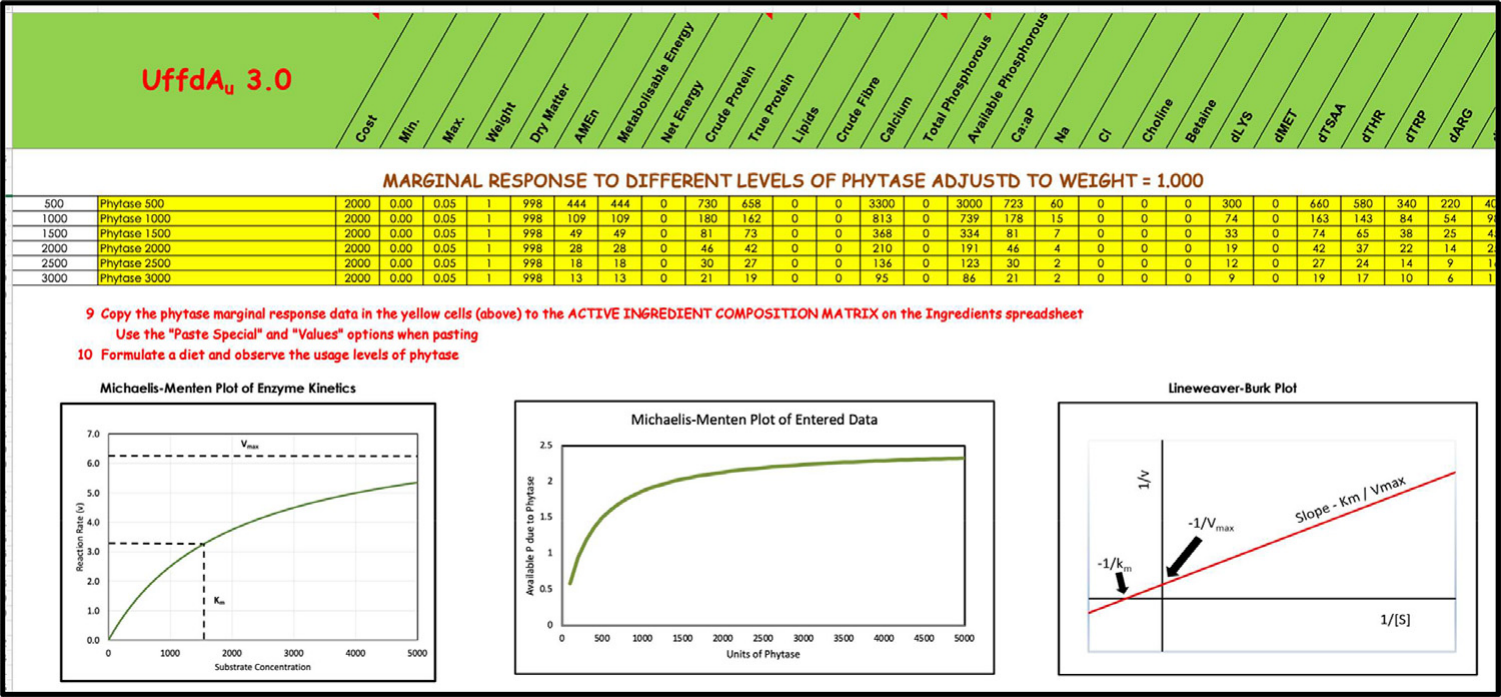
A portion of the UffdAu.xlsm “Enzymes” worksheet to create the step function for simulating nonlinear responses using a linear program.

The Enzymes Worksheet is used to demonstrate how responses to enzymes can be added to linear feed formulation models. A linear approximation of enzyme kinetics is used to determine how much of each nutrient is liberated by different enzyme levels (Figure 6).

The Coefficients Worksheet can be used to update amino acid concentration and digestibility values separately. Results are automatically transferred to the Ingredients worksheet.

The Digestibility Worksheet calculates protein digestibility from amino acid digestibility values. It also contains calculations for nonstarch polysaccharide digestibility values.

The N to TP Factors Worksheet shows how measured amino acid nitrogen to protein conversion factors are used to calculate the true protein content of feed ingredients. Factors for choline and betaine in feed ingredients are included since they are metabolically converted to sarcosine and then glycine.

The AME_n_ Calc Worksheet shows how crude apparent metabolizable energy values are calculated based on the Weende Method of feed ingredient evaluation. AME_n_ values are calculated based on Crude Protein, Crude Fat, Crude Fibre, etc. (WPSA, 1989).

The Composition Charts Worksheet shows the relationship and comparison between the 19th Century Weende Method (proximate analysis) and modern chemical concepts and techniques, labeled the Armidale Method.

The Weende v Armidale Worksheet compares analyzed values for 12 example Australian feed ingredients for 19th vs. 21st Century analytical techniques.

The ME Calc Worksheet shows how ME values are calculated based on metabolic fuel values of ingredient compositions (Kleiber, 1975). The NE Calc Worksheet shows how NE values are calculated based on ATP generated by the feed ingredient components.

The Ingredient Energy Worksheet shows a comparison of AMEn, ME, and NE values for the 12 example Australian feed ingredients.

The energy needs worksheet shows a comparison of AME_n_, ME and NE values for 22 example feeds formulated with the 12 example Australian feed ingredients.

## RESULTS AND DISCUSSION

Students are first taught to formulate feeds with 2 ingredients (perhaps wheat and soybean meal) and 2 nutrients (protein and energy). They enter a new ingredient matrix and requirement restrictions and view the least-cost solution. Subsequent lessons introduce more ingredients and nutrients and advanced features as they are discussed in class lectures. Finally, the Solver routine of Excel has options that perform sensitivity analysis to determine shadow prices for ingredients and nutrients. Shadow price calculation enable students to learn techniques of parametric cost and nutrient ranging. They can observe how changing ingredient prices and nutritional minimums and maximums affect formula prices and ingredient usage.

UffdAu can also be used to set up multi-blending problems where 2 or more feeds are solved simultaneously. Multiblending is used to determine in which formula to use an ingredient available in limited amounts. After students have become familiar with the principles of feed formulation using Excel they can more easily learn to use much more complex commercial formulation software.

The User-friendly Feed formulation program (Pesti and Miller, 1988) was originally created because commercial feed formulation programs were complex, expensive, and had steep learning curves. Excel is an ideal platform to begin teaching feed formulation since most tertiary students already own a copy and are familiar with using it. The students do not have to learn to use a new program and feed formulation at the same time. And they can see exactly what the workbook does simply by clicking on the various cells. Besides cost, those are probably the biggest advantages UffdAu has over other software. It has been helpful in initial lessons where students input values into the worksheet to calculate vitamin/trace mineral premix dilution techniques. This helps students re-familiarize themselves with using Excel and vitamin and trace mineral names and feeding levels.

Earlier versions of UffdA have been adapted by small poultry producers needing to formulate only a few diets. The workbook is easily adaptable to other monogastric species. For instance, versions are available in English, Chinese, French, Spanish and Portuguese for formulating rabbit diets (Gidenne, 2023). Besides UffdAu being free, it gives the same answers as any linear programming tool (https://www.poultryhub.org/research/resources-for-researchers/uffdau).

Progress in nutritional sciences has generally followed very closely behind progress in analytical chemistry. The exception seems to be the continued use of proximate analysis for feed evaluation and feed energy content estimation. Acceptance of updated systems across the nutritional sciences has been hampered by tradition and regulations based on the old system (Krul, 2019). The continued use of the “*inefficient, outdated, and still used*” feed formulation model described by Oviedo-Rondon (2014) should be viewed as a great opportunity by students and practicing nutritionists. New analytical data based on modern chemical concepts (Noh et al., 2020) is continually added to feed formulation models. New, faster, and relatively inexpensive methods are being applied. New near infra-red spectroscopy (**NIRS**) methods are being developed for non-starch polysaccharides and more specific non-starch polysaccharides such as arabinoxylans because of the possibility of adding exogenous xylanase to feed (Nieto-Ortega, 2022). Still, as long as energy estimation is based on crude measures, the resulting metabolizable, net, or similar, energy estimates will necessarily remain crude, and lack precision.

The real problem with using linear programming to determine least-cost poultry feeds is that it assumes that the most economical levels of nutrients is known. If the most economical levels of nutrients are known, then least-cost diets are also profit maximizing ones. Unfortunately, the most economical levels of nutrients are seldom known. When nutrient requirements for poultry are determined for maximum performance, profit-maximizing levels are not known, since they change with prices. Turkey breeding companies sometimes give recommendations for standard diets for normal birds, and high-density diets for high yield birds that are closer to, but still not, necessarily, profit maximizing as prices change. Producers must compare the costs of the diets and expected returns from the sale of the turkeys (Anonymous, 2022; Alonzo, 2023). The biology behind turkey nutrition and growth is the same as that behind broiler chicken nutrition and growth, yet the application in terms of nutritional requirements has been different. Formulation techniques are beginning to include more comprehensive models that include the economics of the input and output relationships of turkeys and chickens (Elwinger et al., 2016; Pesti and Choct, 2023). It is important that students understand the techniques and applications of feed formulation so they are prepared to apply more advanced techniques in the future.

In the past, the measures of feed formulation efficiency have typically been growth rates and feed utilization efficiency. In the future, measures of efficiency should include how poultry meat and egg production contribute to 3 main ecological cycles; the carbon, nitrogen and water cycles. The great success of the modern commercial poultry industry began because of its’ very positive relationship with the farming ecology of the Southeastern United States in the 1930s (Sawyer, 1971). Poor, depleted soils made traditional types of farming unsustainable. The introduction of poultry farming, with much imported grain, led indirectly to the re-establishment of organic topsoils and subsequently, the development of lucrative, sustainable, cow-calf pasture operations. Maize sequestered large amounts of atmospheric carbon and soybeans sequestered large amounts of atmospheric carbon and nitrogen. The sequestered carbon and nitrogen produced food, by-product feed ingredients, and manure that acumuated in soils. Perhaps most importantly, manure added to the soil is both a carbon sink and has great water-holding capacity. Water-holding capacity decreases water run-off, erosion and reduces the requirement for consistent rain. UffdAu provides students with a tool to promote understanding of the biology and economics of just part of the poultry production ecological system. For instance, the costs of different protein levels versus the cost of manure disposal or fertilizer value can be explored. Whilst current feed formulation techniques may well be outdated and inefficient, understanding the principles of feed formulation is an important part of mastering (or grasping) the overall impact of feed utilizations’ role in meat and egg production, business profitability and farming ecology.

## CRediT authorship contribution statement

**G.M. Pesti:** Conceptualization, Methodology, Formal analysis, Validation, Investigation, Writing – original draft, Writing – review & editing. **M. Choct:** Conceptualization, Writing – review & editing. **P.V. Chrystal:** Conceptualization, Writing – review & editing. **M.R. Bedford:** Conceptualization, Methodology, Writing – review & editing. **A.F. Moss:** Conceptualization, Writing – review & editing.
